# The application of the modified surgical wound dressing in wound care after tracheotomy

**DOI:** 10.1051/rmr/160001

**Published:** 2017-04-24

**Authors:** Mei Feng, Ying Wu, Jing Zhu, Xiaoling Wu

**Affiliations:** West China Hospital, Wainanguoxuexiang 65, Chengdu, Sichuan PR China

**Keywords:** Tracheotomy, wound infection, wound closure time, patient comfort, dressing

## Abstract

*Introduction*: This study was performed to observe the efficacy of a modified surgical wound dressing applied as part of decannulation wound care after tracheotomy.

*Methods*: Eighty-four patients were randomly allocated into a traditional care group, a surgical wound dressing group, and a modified surgical wound dressing group. Each group comprised 28 patients. The following outcomes were observed and analyzed: infection rate, wound closure time, dressing change frequency, cost of wound care, and patients' comfort.

*Results*: The infection rate, wound closure time, dressing change frequency, and cost of wound care were much higher in the traditional care group than in the surgical wound dressing group and modified surgical wound dressing group. The study data indicated that patients in the modified surgical dressing group felt more comfortable than those patients in the other two groups. The differences among the three groups were statistically significant (*P* < 0.05).

*Discussion*: The design of the herein-described modified surgical wound dressing is based on a butterfly shaped adhesive and mirrors the advantages of a modern surgical wound dressing. Its shape is suitable for the physiological structure of the neck, making it more comfortable to use. Aseptic packaging and a high degree of adhesiveness guarantee continuous fixation and pulling. At the same time, the design of the dressing decreases the chance of infection.

## Introduction

Tracheotomy is an important method of maintaining airway patency in critically ill patients. The endotracheal tube is removed when the patient's disease status has stabilized, but this leaves an open wound in the patient. Adhesive dressing is required to pull the edges of the wound together and thus promote wound healing. In traditional practice, a fabric adhesive material is cut into a butterfly shape. Gauze is placed over the wound, and the fabric adhesive is pasted onto the wound and over the gauze after being heated by a Bunsen burner. Nevertheless, this is an inefficient technique because the fabric adhesive is not sticky enough, and it readily detaches when the neck moves. Moreover, ordinary gauze becomes stiff when contaminated by secretions and sputum because of the characteristics of the material, making it uncomfortable for the patients and increasing the risk of infection. Bandages [4], hydrocolloid dressing [3], and foam dressing [2] have reportedly been used in wound care after extubation. However, bandages are small and not suitable for larger wounds. Additionally, hydrocolloid dressing and foam dressing are expensive and not sticky enough to pull the wound together. To promote wound healing, decrease the chance of infection, and increase patients' comfort, we applied a modified surgical wound dressing based on the butterfly shaped adhesive traditionally used for wound care after tracheotomy. Positive outcomes were obtained.

## Methods

### Sample

This study was performed at a regional teaching hospital. In total, 84 patients (53 male, 31 female) who underwent tracheotomy from March 2015 to February 2016 were included. Sixty-eight patients were diagnosed with chronic obstructive pulmonary disease, four with broncho-stenosis, and 12 with pulmonary infection. Thirty-six patients were treated with metal tubes and 58 patients were treated with silicon tubes. The ages ranged from 36 to 84 years (average, 61 ± 0.38 years). The patients were randomly allocated into a traditional care group, surgical wound dressing group, and modified surgical wound dressing group by a computer program (SPSS 19.0; IBM Corp., Armonk, NY, USA). There were no significant differences in the patients' baseline characteristics, including age, sex, diagnosis, tube material, wound size, and catheter dwelling time (*P* > 0.05).

The inclusion criteria were a conscious ability to verbally or literally express feelings and no sign of infection upon tube removal. The patients voluntarily participated in the research, and informed consent was obtained from all patients.

The exclusion criteria were unconsciousness and an inability to express feelings, diabetes or cachexia, and evidence of wound infection upon tube removal. The indications of wound infection were redness, swelling, and infiltration of the wound site or a body temperature of >37.3 °C with no other clear sources of infection. The wounds were assessed by a qualified wound care nurse who did not participate in the research.

### Procedure

Wound care after extubation was performed according to the hospital standard. The wound was firstly cleaned with normal saline, and the skin was then disinfected with 75% ethyl alcohol. The edges of the wound were then pulled together, and the dressing was pasted onto the wound.

**Figure 1:**
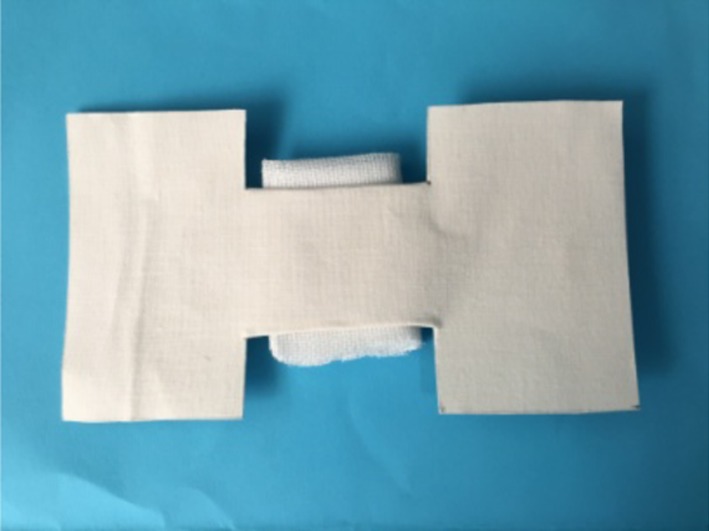
Traditional adhesive.

In the traditional care group, the fabric adhesive was cut into a butterfly shape with a tape length of 14 cm and wing width of 8 cm (Figure 1). The center of the tape was about 5 cm wide. The wound was cleaned and covered with sterilized gauze. The tape was heated by a Bunsen burner and pasted onto the gauze while the wound was pulled together with hands.

**Figure 2:**
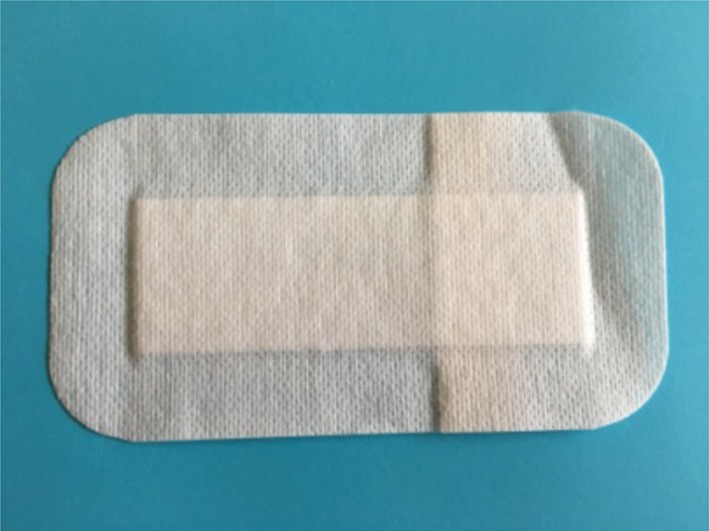
Surgical wound dressing.

In the surgical wound dressing group, a surgical wound dressing from Paul Hartmann Trade Co., Ltd. (Shanghai, China) was used. The size of the dressing was 15 × 8 cm^2^ (Figure 2). The wound was firstly cleaned and then covered by the dressing while the wound was pulled together with hands.

**Figure 3:**
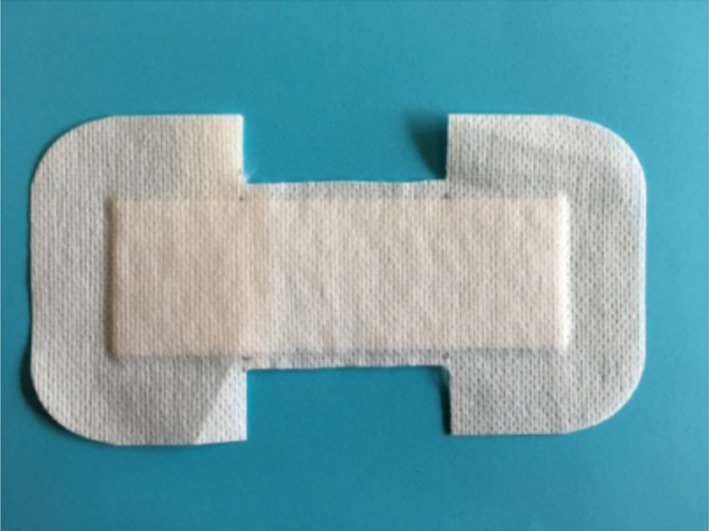
Modified surgical wound dressing.

In the modified surgical wound dressing group, the same dressing as used in the surgical wound dressing group was utilized, but the dressing was cut into a butterfly shape with sterilized scissors (Figure 3). The center was approximately 5 cm wide. The wound was firstly cleaned and then covered by the dressing while the wound was pulled together with hands.

### Primary outcome measure

Wound assessments and dressing changes in the different groups were performed by the same wound care nurse. Wound infection was used as the primary outcome. The infection was assessed and measured with a ruler when the dressing was being changed and graded as follows [5]. Grade 0 infection: no sign of infection such as redness, swelling, or pain. Grade I infection: *d* < 0.5 cm diameter of redness and/or swelling affecting the wound site and skin. Grade II infection: *d* > 0.5 cm diameter of redness and/or swelling affecting the wound and site skin. Grade III infection: redness and/or swelling affecting the wound and site skin accompanied by purulent secretion.

### Secondary outcome measures

The secondary outcome measures were the wound closure time, dressing change frequency, cost of wound care, and patients' comfort.

#### Wound closure time

A wound was defined as closed if it met the following criteria. At first, the edges of the wound were closed and no crack was visible on the surface of the skin. Second, no air or sputum was expressed from the wound when the patient coughed.

#### Dressing change frequency

The dressing was changed weekly if it was not contaminated or wet. If the dressing was contaminated by sputum or secretions, or if pain was expressed by the patient, the dressing was changed immediately. The dressing was also changed when it contained blood or pus.

#### Cost of wound care

The cost of wound care was calculated by the dressing cost, dressing change fees (18 Yuan per time as priced by the local health department for the medical workers' labor, tape and gauze).

#### Patients' comfort

Comfort was defined by the patients' own feelings, either verbally or literally expressed, regarding the security of the dressing, wound pain severity, and neck mobility. Pain was evaluated by the Wong–Baker faces pain rating scale (a score of 0–4 was considered comfortable, while a score of 6–10 was considered uncomfortable). Neck mobility was assessed by visual judgment and assessment of the range of flexion by turning the neck to the right and left (a range of 45° in both directions with no expression of pain by the patient was considered comfortable, while a range of <45° was considered uncomfortable).

#### Statistical analysis

Data were recorded using Microsoft Excel software and analyzed using SPSS 19.0. The differences between the groups were compared using the chi-square test.

## Results

### Traditional care group vs. surgical wound dressing group

In the traditional care group, seven patients developed a grade I infection, five developed a grade II infection, and two developed a grade III infection. The infection rate was 50%. The two patients with a grade III infection remained in the study because the manifestations of infection were redness and swelling on the wound tissues along with slight purulent secretion. These were local infections; the patients' hemograms were normal. Therefore, no extra antibiotic treatments were given to these two patients. However, more effort was given to the patients' wound care. In comparison, three patients in the surgical wound dressing group developed a grade I infection; the infection rate was 10.7%. The infection rate in the surgical wound dressing group was much lower than that in the traditional care group, and the patients' comfort level was higher. The differences were statistically significant (*P* < 0.05). Moreover, the wound closure time, dressing change frequency, and cost of wound care were significantly lower (Table 1).

**Table 1:** Comparison between traditional and surgical wound dressing groups.

	Infection Rate (case)	Patient's comfort (case)	Average closing time (d)	Dressing change times	Average wound care cost (RMB: Yuan)
		Comfortable			
Traditional Group *n* = 28	14	3	7.57 ± 1.73	4.07 ± 0.86	73.29 ± 15.44
Surgical wound dressing group *n* = 28	3	18	4.71 ± 0.40	1.82 ± 0.39	46.63 ± 9.98
*t* or *X*^2^	8.446	14.933	8.255	12.638	7.673
*P*-Value	0.004	0.000	0.000	0.000	0.000

### Surgical wound dressing group vs. modified surgical wound dressing group

Two patients developed a grade I infection in the modified surgical wound dressing group. The infection rate was 7.1% in this group. There were no significant differences in the infection rate, wound closure time, dressing change frequency, or cost between the modified surgical wound dressing group and surgical wound dressing group (*P* > 0.05). However, more patients felt comfortable in the modified surgical wound dressing group. This difference was statistically significant (*P* < 0.05) (Table 2).

**Table 2:** Comparison between surgical wound dressing group and modified surgical wound dressing group.

	Infection Rate (case)	Patient's comfort (case)	Average closing time (d)	Dressing change times	Average wound care cost (RMB: Yuan)
		Comfortable			
Surgical wound dressing group *n* = 28	3	18	4.71 ± 0.60	1.82 ± 0.39	46.63 ± 9.98
Modified surgical wound dressing group *n* = 28	2	27	4.43 ± 0.57	1.61 ± 0.49	42.06 ± 12.49
*t* or *X*^2^	0.000	7.24	1.794	1.794	1.513
*P*-Value	1	0.007	0.074	0.079	0.136

### Modified surgical wound dressing group vs. traditional care group

The infection rate, patients' discomfort, wound closure time, dressing change frequency, and cost were significantly lower in the modified surgical wound dressing group than in the traditional care group (*P* < 0.05) (Table 3).

**Table 3:** Comparison between traditional wound dressing group and modified surgical wound dressing group.

	Infection Rate (case)	Patient's comfort (case)	Average closing time (d)	Dressing change times	Average wound care cost (RMB: Yuan)
		Comfortable			
Traditional Group *n* = 28	14	3	7.57 ± 1.73	4.07 ± 0.86	73.29 ± 15.44
Modified surgical wound dressing group *n* = 28	2	27	4.43 ± 0.57	1.61 ± 0.49	42.06 ± 12.49
*t* or *X*^2^	10.588	37.979	9.123	13.153	8.322
*P*-Value	0.001	0.000	0.000	0.000	0.000

## Discussion

### Significantly lower infection rate and wound closure time and higher patient comfort in modified surgical wound dressing group

The butterfly shaped adhesive provides the neck with more flexible mobility. Whereas, the material of a traditional butterfly shaped adhesive is fabric, which cannot effectively absorb wound secretions. Therefore, gauze must also be used. But the gauze dries very quickly and therefore cannot maintain moisture in the wound. The dry environment is not conducive to growth of epithelial tissue. The gauze adheres to the scab of the wound, which can cause mechanical damage when the dressing is changed. This in turn decreases the patient's comfort. The wound readily becomes contaminated when the gauze detaches from the wound [1]. This is because the gauze and the adhesive tape are not completely fused when the patient's neck is moving, and the gauze readily drops off. The tape then comes into direct contact with the wound but does not meet the standard of sterilization. Therefore, the tape increases the chance of infection. This leads to an extension of the wound closure time and an increase in the patient's discomfort. The gauze dropped off in two patients in the present study.

Surgical wound dressing comprises several layers. The bottom liner is coated with self-adhesive polystyrene and an absorbent cotton pad. It has a strong tenacity, and the cotton pad can effectively absorb secretions, thus keeping the wound clean and avoiding infection. But the dressing is square in shape; such dressing is usually applied in surgical or superficial wound care. Because the neck requires a high degree of mobility, patients feel tension and limited motion when the neck is fixed with such a dressing. In the present study, 10 patients felt uncomfortable in the surgical wound dressing group because the tension in the neck adversely influenced neck motion and bending.

The modified surgical wound dressing provides a combination of the advantages of both the butterfly shaped adhesive and the surgical wound dressing. It guarantees securement, good absorption, and flexible neck movement, thus increasing the patient's comfort.

### Decreases in dressing change frequency, cost, and nurse workload with modified wound dressing

Traditional butterfly shaped tape is not adhesive enough and does not effectively pull the wound together. When the patient coughs, sputum can be easily expressed from the wound. When the tape becomes wet, the tape loses viscidity. Thus, the tape can easily become detached and requires frequent changes. Patients in the traditional care group of the present study required an average of four dressing changes. The average cost was 72 Yuan based on a dressing change fee of 18 Yuan per time (gauze and tape were included). This increased both the cost of wound care and the nurse's workload (the nurse spent an average of 15 min on wound care, including equipment preparation). Both the surgical wound dressing and the modified surgical wound dressing were adhesive and pulled the wound together tightly. The sputum was merely coughed out from the wound. Therefore, the need for dressing changes decreased because the dressing did not become wet. If the wound does not become infected, it can close in 3–5 days. Therefore, the patients in the modified surgical dressing group only required one to two dressing changes. The cost for these changes was 25.6 Yuan per time, including the dressing fee of 7.6 Yuan and dressing change fee of 18.0 Yuan. This cost was obviously lower than that in the traditional care group, and the time spent by the nurse on the dressing change also decreased.

## Conclusion

Critically ill patients have weak immune systems. The artificial airway and invasive treatment are the main sources of infection [6]. Nonstandard nursing care of the wound after tracheotomy can also cause infection. Therefore, it is vital to choose an appropriate dressing for the wound care. The herein-described modified surgical wound dressing maintains the advantages of both a butterfly shaped adhesive and surgical wound dressing. It guarantees security of the dressing and allows for flexible neck movement, thus increasing the patient's comfort and decreasing the wound closure time. The aseptic packaging and good viscidity provide continuous protection of the wound. Additionally, the modified surgical wound dressing is complete in that the cotton pad does not become detached from the self-adhesive polystyrene. This decreases the chance of infection compared with the traditional dressing, from which the gauze can easily drop off.

## Limitations

This study was not double-blinded; both the nurses and patients could distinguish the different groups based on the appearances of the dressings. This is because the study was performed to test the efficacy of the three differently designed dressings. This lack of blinding may have led to bias in the patients' expression of feelings and the wound care nurse's judgment if an attempt was made to satisfy the research nurses. To decrease this bias, quantitative instruments were used to assess the patients' pain and neck mobility. Additionally, a clearly defined standard was used to assess the severity of infection. Moreover, the wound nurse was not involved in the research. A larger sample size is required to evaluate these findings in a future study.

## Conflict of interest

All authors certify that they have no financial conflict of interest..
